# Barbed and Non-Barbed Suture Materials for Ventral Hernia Repair: An Experimental Study

**DOI:** 10.3390/jcm14093139

**Published:** 2025-05-01

**Authors:** Georgy B. Ivakhov, Svetlana M. Titkova, Mikhail V. Anurov, Aleksandra A. Kalinina, Konstantin I. Shadin, Vladimir V. Suglob, Andrey V. Andriyashkin, Alexander V. Sazhin

**Affiliations:** 1 Institute of Surgery, Pirogov Russian National Research Medical University, Moscow 117513, Russia; ivakhovsurg@yandex.ru (G.B.I.); stitkova63@yandex.ru (S.M.T.); anurov-m@yandex.ru (M.V.A.); konstantin.shadin@gmail.com (K.I.S.); dr@vsuglob.ru (V.V.S.); dr.andriyashkin@gmail.com (A.V.A.); sazhin-av@yandex.ru (A.V.S.); 2 City Clinical Hospital No. 1 Named After N.I. Pirogov, Moscow 119049, Russia

**Keywords:** barbed sutures, monofilament sutures, ventral hernia repair, mechanical tensile tests, tissue reaction

## Abstract

**Objectives:** The objective of this study was to assess the tissue response and strength of traditional and unidirectional suture materials, depending on the conditions of use and the timing following implantation. **Methods:** Eighty male Wistar rats were randomly assigned to four groups depending on the suture used: unidirectional absorbable V-loc^TM^ 180 or non-absorbable V-loc^TM^ PBT and traditional absorbable Maxon^TM^ or non-absorbable Novafil^TM^. Three and six weeks following the closure of the abdominal wall defect (AWD) and subcutaneous suture implantation at the withers according to group assignment, 10 animals from each group were euthanized for implanted sutures mechanical testing and histological examination. **Results:** The inflammatory reaction in the AWD closure area was maximal and significantly different from the subcutaneous implantation by week 3 for all groups. At six weeks, the tissue reaction did not depend on the place of implantation. However, four rats from the Maxon^TM^ group demonstrated suture failure with diastasis formation. Non-absorbable barbed sutures exhibited an absence of suture failure and the maximum scar thickness. Both intact absorbable materials (V-Loc^TM^ 180 and Maxon^TM^) exhibited a significant breaking strength margin over the non-absorbable. By week 6, the preserved strength of the V-loc^TM^ 180 sutures at the AWD was 33% (15–58%), and under the skin—49.7% (48–59%) (*p* = 0.005). For Maxon^TM^, these values were 38% (35–48%) for the AWD and 44% (34–49%) for the subcutaneous implantation. **Conclusions:** Absorbable and non-absorbable suture materials, depending on the conditions and timing of implantation, cause various tissue reactions which could affect the wound healing and the number of postoperative complications.

## 1. Introduction

In recent years, there has been a notable increase in the proportion of minimally invasive surgical procedures employed in the treatment of patients with postoperative ventral hernias. This is largely attributed to the advantages of a shorter recovery period and a reduced incidence of surgical wound infection, as evidenced by a growing body of literature [1,2,3,4,5].

Minimally invasive hernia repair entails the suturing of the aponeurosis under conditions of limited visibility, and therefore requires devices to close the defect, which facilitate the tissue approximation and obviate the necessity for tying knots. Such devices include unidirectional knotless monofilament surgical sutures. Following the Food and Drug Administration (FDA) approval of these sutures for soft tissue approximation in 2005, numerous surgeons conducted research to assess the safety and effectiveness of these devices in various surgical procedures [6,7,8,9]. Barbed sutures are currently in use for a variety of indications, including cosmetic plastic surgery [10], obstetrics and gynecology [11,12], gastrointestinal surgery [13], urology [14], and pediatric and bariatric surgery [15,16]. Clinical studies demonstrated that the utilization of unidirectional barbed sutures significantly reduced the time required for wound closure and the tissue injury during the procedure and also provided satisfactory results for both surgeons and patients [17]. Although complications associated with the use of certain types of unidirectional sutures have been described [18,19], these sutures are believed to be safe and effective in various types of surgical procedures.

Currently, a variety of barbed sutures from different manufacturers are available on the market. At the same time, all the forementioned authors highlight the lack of a standardized technique or recommendations on the choice and specifics of using unidirectional sutures for specific surgical interventions.

Therefore, we conducted an experimental study for the comparison of various suture materials utilized in prosthetic hernia repair for fascial defects closure. The objective of this study was to assess the tissue response and strength of the suture material (traditional and unidirectional), depending on the conditions of use and the timing following implantation.

## 2. Materials and Methods

### 2.1. Animals

The experimental study was conducted from March to September 2022 in 80 outbred male Wistar rats, aged 6 months and weighing 350–400 g. The rats were maintained in standard controlled conditions at the vivarium, with a 12 h light cycle and free access to water and food. All experiments involving animals were conducted in accordance with the international guidelines for the ethical treatment of laboratory animals [20]. The study protocol was approved by the Pirogov University Institutional Animal Care and Use Committee (approval number: 25|2021; date of approval: 10 December 2021).

### 2.2. Study Design

The animals were randomly assigned to one of four groups, with 20 rats in each group. Group 1 was treated with absorbable unidirectional suture material V-loc ^TM^ 180 3-0 (Medtronic Covidien, Mansfield, MA, USA), which is composed of a copolymer of glycolic acid and trimethylene carbonate. Group 2 was treated with non-absorbable suture material V-loc^TM^ PBT 3-0 (Medtronic Covidien, Mansfield, MA, USA), which is composed of polybutester copolymer butylene terephthalate (PBT) and polytetramethylene glycol ether. Group 3 was treated with traditional absorbable monofilament suture material Maxon^TM^ 3-0 (Medtronic Covidien, Mansfield, MA, USA), which is composed of polyglyconate, glycolic acid copolymer, and trimethylene carbonate. Finally, group 4 was treated with traditional non-absorbable monofilament suture material Novafil^TM^ 3-0 (Medtronic Covidien, Mansfield, MA, USA), composed of polybutester copolymer butylene terephthalate and polytetramethylene glycol ether.

### 2.3. Study Outcomes

The primary endpoint was to assess tensile strength of the suture material and to characterize, by histopathological analysis, the tissue response to four types of studied sutures depending on the surface textures (barbed and non-barbed), physical properties (absorbable and non-absorbable), conditions of use, and the timing following implantation.

The secondary endpoint was to assess the rate of suture failure in studied groups.

### 2.4. Surgery

**Figure 1:**
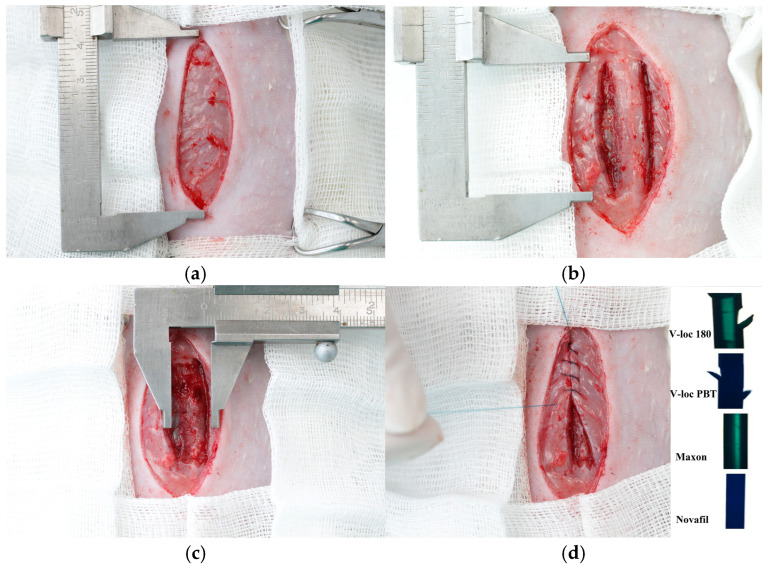

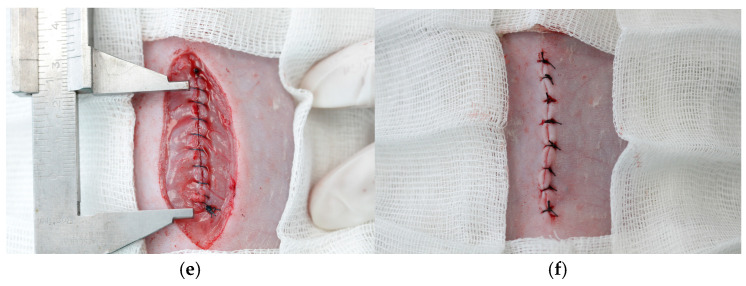
A myofascial defect creation: the skin incision (**a**), the dissection of the anterior layer of the rectus sheath on both sides of the linea alba (**b**), the partial resection of the medially located fibers of the rectus abdominis muscles (**c**), suturing of the formed defect with 1 of the 4 types of sutures under study (**d**,**e**), and the closed skin with interrupted sutures (**f**).

**Figure 2:**
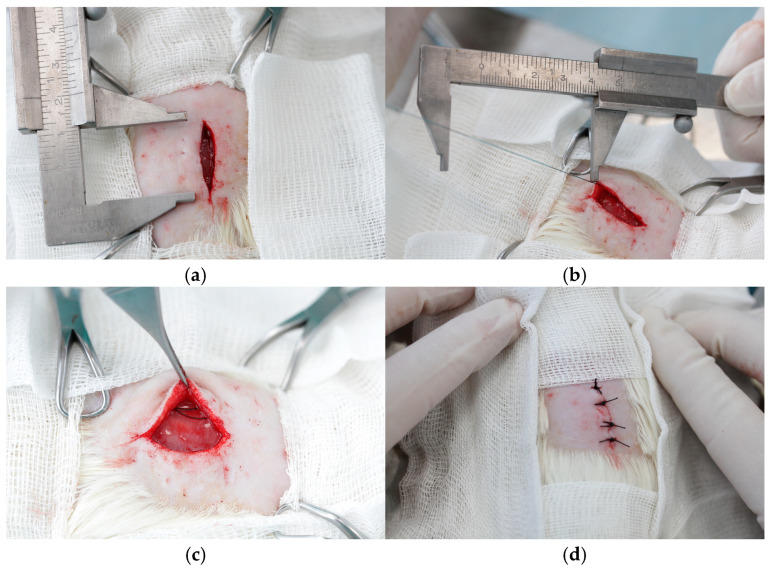
The subcutaneous implantation of the suture on the back: the 2 cm long paravertebral skin incision (**a**), the placing and fixation of the suture material into the formed subcutaneous pocket (**b**,**c**), and the skin closure with interrupted silk sutures (**d**).

All surgical procedures were conducted under general anesthesia in sterile conditions. The induction anesthesia of 4% Isoflurane («Laboratories Karizoo, S.A.», Barcelona, Spain), while Zoletil 100 (Virbac, Carros, France), administered at a dose of 3 mg/100 g body weight via intramuscular injection, was utilized for the maintenance of anesthesia. Following the shaving and treatment of the anterior abdominal wall (AAW) with an antiseptic solution, a 5 cm long skin incision was performed (Figure 1a). Subsequently, a myofascial defect was created by dissection of the anterior layer of the rectus sheath on both sides of the linea alba for a length of 30 mm (Figure 1b), followed by separating the rectus muscle from the posterior layer of the rectus sheath. Next, the rectus muscles were separated from the anterior fascial layer and the medially located fibers of the rectus abdominis muscles (approximately 5 mm on each side) were partially resected (Figure 1c). The linea alba was completely removed for 30 mm, after which the formed defect was sutured with 1 of the 4 types of sutures under study, continuously using the small bite suturing technique (Figure 1d,e). Subsequently, the skin was closed with interrupted sutures (Silk 3-0) (Figure 1f), after which the rat was turned over and a suture with a length of at least 6 cm, corresponding to the suture used to close the defect, was implanted subcutaneously on the back above the scapulae. To do this, following shaving and antiseptic treatment, a 2 cm long skin incision was made paravertebrally (Figure 2a), a subcutaneous pocket was formed bluntly, and the suture material was placed and fixed with a single suture to the underlying muscle (Figure 2b,c). After placing the free end of the suture the skin was closed with interrupted sutures (Figure 2d).

### 2.5. Follow-Up

Three and six weeks following the surgical procedure, 10 animals from each group were euthanized by an anesthetic overdose for mechanical testing of the implanted sutures and for histological examination. To perform the mechanical tests, the sutures were carefully extracted from the AAW and from the implantation site under the skin without damaging the scar.

### 2.6. Mechanical Tensile Tests

All mechanical tensile tests were conducted on a single-column universal materials testing machine “TA.XTplus Texture Analyser”, manufactured by Stable Micro Systems Ltd. (Godalming, UK). The stretching diagrams were processed in semi-automatic mode using Exponent Stable Micro Systems Version 6.1 for Windows 10 software. Maximum load to failure in Newtons (N) was determined on load–displacement curves for the sutures.

**Figure 3:**
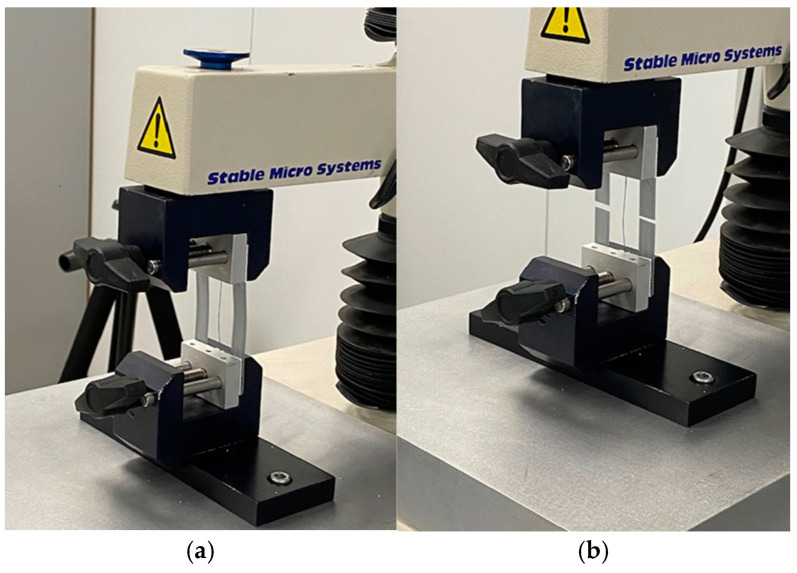
The preparation of the sutures for mechanical testing: the fixation of the frame in grips (**a**) and cutting the frame (**b**).

To perform mechanical tensile tests until rupture, all samples of suture material extracted from the AAW and the implantation site under the skin on the back were fixed in specially prepared frames using epoxy glue. Then, the frames were fixed in standard grips (Figure 3a) and cut on both sides of the sample (Figure 3b).

The samples were clamped using the strip method, with a 30 mm clamp spacing and subjected to a tensile strain rate of 0.5 mm per second until rupture. From each group, 6 sutures extracted from the AAW and 6 sutures extracted from the subcutaneous pocket at the withers were used for mechanical tests. Intact samples of the corresponding suture material served as a control. For this purpose, 4 blisters of suture material of each type were used (V-Loc ^TM^ 180 (Medtronic Covidien, Mansfield, MA, USA), V-Loc^TM^ PBT (Medtronic Covidien, Mansfield, MA, USA), Maxon^TM^ (Medtronic Covidien, Mansfield, MA, USA), and Novafil^TM^ ((Medtronic Covidien, Mansfield, MA, USA)). Two samples were cut out of each blister for testing (eight samples for each type of suture material). The needles were removed from intact sutures, and the sutures were weighed on the Sartorius Research analytical scales with an accuracy of 0.1 mg. The obtained values were recalculated in g/m. The diameter of the studied sutures was determined from microphotographs taken with a Moticam 3000C microscopic camera (Motic Europe, Barcelona, Spain) at ×100 magnification using the Motic Images Advanced 3.2 program.

All mechanical tests were performed by two researchers experienced in biomechanical testing.

### 2.7. Histopathology

Four animals from each group were randomly selected for histological analysis. The anterior abdominal wall was excised and transferred to a cork board, on which the specimen was stretched using needles under slight tension. Subsequently, a 25 mm wide strip, passing through the area of the postoperative scar, was cut out from each anterior abdominal wall in a transverse direction relative to the midline. Abdominal wall thickness was measured with a digital thickness gauge using a polished disk with a diameter of 31 mm under pressure of 1 KPa. The tissue samples from the abdominal wall and the site of suture implantation on the back were harvested and fixed in 10% buffered formalin solution. The fixed samples were embedded in paraffin, sectioned at 5 µm, and stained with hematoxylin and eosin stain. Each section then was examined by two pathologists in a blinded fashion under light microscopy to assess the relative degree of tissue reaction surrounding each suture type using a modified scoring system (Table 1).

### 2.8. Statistical Analysis

The statistical analysis was conducted using the Statistica StatSoft 13.0 package. For continuous variables, the data with a normal distribution were presented as the mean value with standard deviation, for data with a distribution different from normal as median values with the interquartile range [IQR]. The normality of the data was evaluated using the Shapiro–Wilk test. For paired comparison of continuous variables with a normal distribution, the t-test was used for independent samples, and Mann–Whitney U-test was used for variables without a normal distribution. Multiple comparisons of independent variables distributed normally were performed using single-factor analysis of variance (ANOVA), followed by a comparison using the Tukey test in case of significant differences between the groups. When comparing several samples of quantitative data with a distribution different from normal, the Kruskal–Wallis criterion was used. If significant differences were found between the groups, post hoc comparisons of mean ranks of all pairs of groups with a Bonferroni adjustment were additionally conducted. The *p* values less than 0.05 were considered statistically significant.

The F test for analysis of variance (ANOVA) was used for sample size calculation. To detect a medium size effect with type I error of 0.05 and power (1-β err prob) of 0.8, 20 rats per group were required.

## 3. Results

### 3.1. Complications

**Figure 4:**
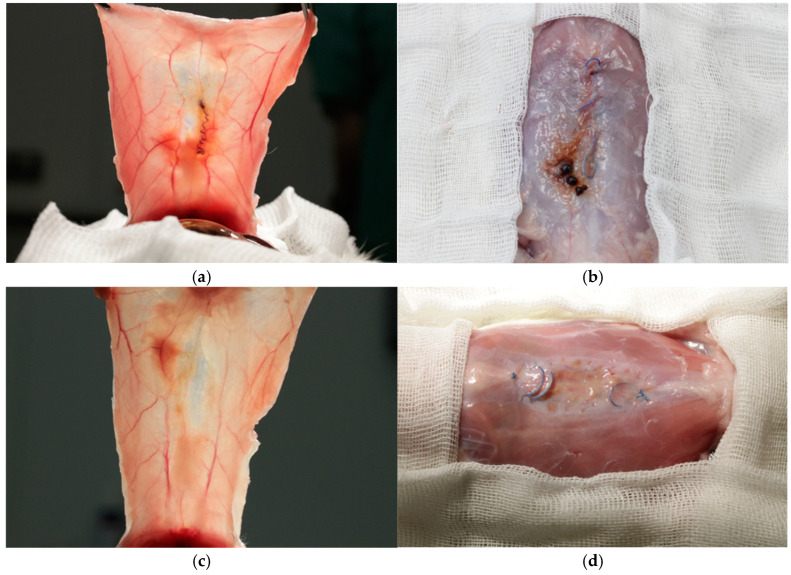
Complications 3 weeks following suturing of myofascial defect: (**a**), infection of V-Loc^TM^ 180 suture, which led to suture failure and diastasis formation; (**b**), hematomas around V-loc^TM^ PBT suture; and suture failure with diastasis formation in Maxon^TM^ (**c**) and Novafil^TM^ (**d**) groups.

All the animals completed the study protocol. No complications were detected at the site of the suture implantation on the back in any group. Three weeks following the suturing of the myofascial defect, a dense postoperative scar was formed in most animals from all groups. In one animal from the group of absorbable unidirectional suture material V-Loc^TM^ 180, microabscesses were detected around the sutures, which led to suture failure and the development of diastasis (Figure 4a). One animal from the V-loc^TM^ PBT group developed hematomas around the sutures without signs of infection and suture failure (Figure 4b). Finally, two animals from group 3 (Maxon^TM^) and one animal from group 4 (Novafil^TM^) demonstrated the suture failure of a myofascial defect and diastasis formation (Figure 4c,d).

**Figure 5:**
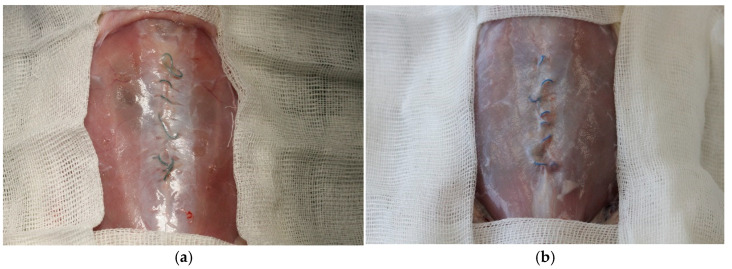

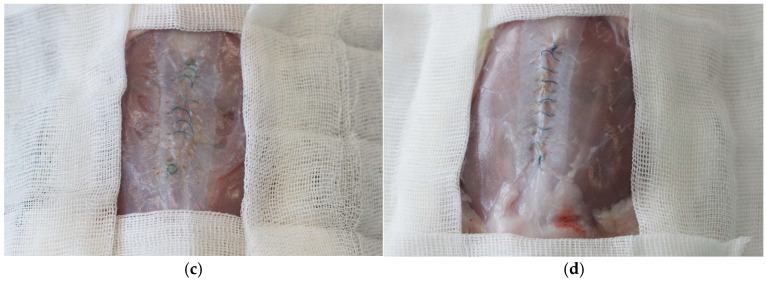
The postoperative scar and the studied sutures 6 weeks following the suturing of the myofascial defect: (**a**), V-Loc^TM^ 180; (**b**), V-Loc^TM^ PBT; (**c**), Maxon^TM^; and (**d**), Novafil^TM^.

Six weeks following the suturing of the myofascial defect, the majority of animals in all groups exhibited complete healing with the formation of a scar, around which sutures were freely located without tension (Figure 5).

**Figure 6:**
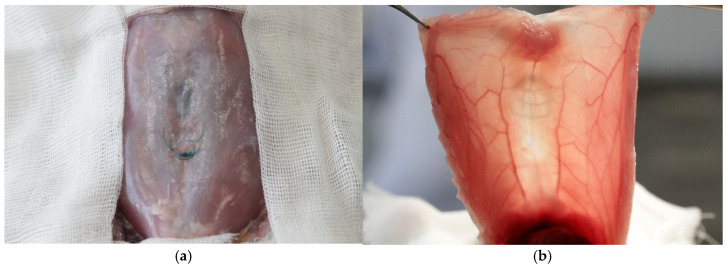
The suture failure (**a**) with the formation of diastasis, (**b**) 6 weeks following the suturing of the myofascial defect in an animal from the Maxon^TM^ group.

Only in the Maxon^TM^ group, the failure of the myofascial sutures with the formation of diastasis was observed in four out of ten operated animals (Figure 6).

The thickness of the formed postoperative scar did not differ between the groups three weeks following the procedure. By week 6, the thickness of the scar increased significantly in the groups of unidirectional sutures (both absorbable V-Loc^TM^ 180 and non-absorbable V-Loc^TM^ PBT), but did not change in the traditional suture material groups, Maxon^TM^ and Novafil^TM^ (Table 2).

The maximum thickness of the postoperative scar six weeks following the procedure was observed in the V-Loc^TM^ PBT group, and was significantly different from the Maxon^TM^ (*p* = 0.02) and Novafil^TM^ (*p* = 0.03) groups.

### 3.2. Structural and Mechanical Properties of Intact Sutures

**Figure 7:**
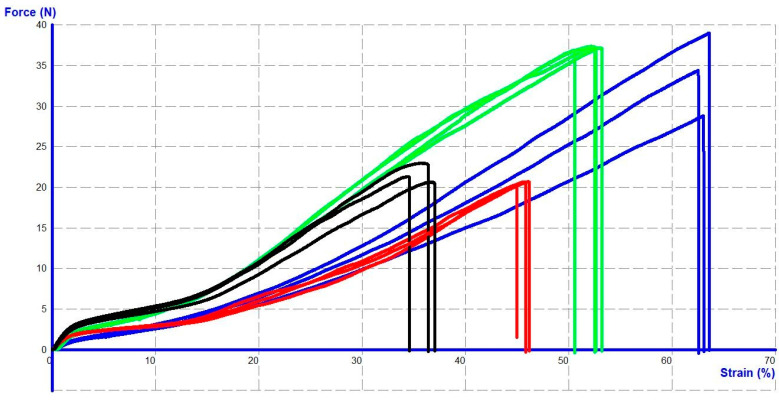
Examples of the force–strain curves for intact sutures: V-loc^TM^ 180 (green), V-loc^TM^ PBT (black), Maxon^TM^ (blue), and Novafil^TM^ (red).

Despite being the same size (USP 3-0), all the studied sutures showed different structural and mechanical characteristics. In particular, the intact samples demonstrated different strength parameters in the mechanical tests, as evidenced by Table 3 and Figure 7.

Both absorbable materials (V-Loc^TM^ 180 and Maxon^TM^) exhibited a statistically significant breaking strength margin over the non-absorbable samples (V-Loc^TM^ PBT and Novafil^TM^). The strength of V-Loc^TM^ 180 significantly exceeded that of Maxon^TM^ (*p* = 0.002).

The weight and diameter of the V-loc^TM^ 180 suture also exhibited the greatest values, 167.9 ± 0.14 mg/m and 0.361 ± 0.002 mm. Novafil^TM^ monofilament sutures had a minimum weight and diameter, 58.6 ± 0.01 mg/m and 0.236 ± 0.001 mm. The weight and diameter of V-loc^TM^ PBT and Maxon^TM^ were 105.6 ± 0.07 mg/m with a suture diameter of 0.313 ± 0.002 mm and 108.7 ± 0.07 mg/m with a suture diameter of 0.295 ± 0.004 mm, respectively. The diameter of the suture “under the barb” of the unidirectional V-loc^TM^ 180 was significantly higher than that of the V-loc^TM^ PBT (0.299 ± 0.001 vs. 0.275 ± 0.012, *p* = 0.019).

### 3.3. The Mechanical Properties of the Sutures Depend on the Location and Timing of Implantation

The results of the mechanical testing of the sutures are presented in Table 4.

Three weeks following the implantation, the V-loc^TM^ 180 sutures extracted from the AAW and from the subcutaneous pocket at the withers demonstrated no differences in strength either among themselves or compared with the intact material. Six weeks following the implantation, the breaking strength of the sutures decreased significantly, both in comparison to the intact sutures and to the 3-week values. The AAW sutures exhibited a more pronounced reduction in strength compared to the subcutaneous sutures. As anticipated, the load to failure of the sutures from the non-absorbable V-loc^TM^ PBT material did not change over time and did not depend on the place of implantation. A significant decline in the tensile strength of the absorbable Maxon^TM^ suture was observed at 6 weeks post-implantation, with no discernible difference in the mechanical properties of the suture depending on the location. At 3 weeks, insignificant differences were observed between the sutures from the AAW and from the subcutaneous pocket at the withers. However, in contrast to the absorbable V-Loc^TM^ 180 suture, the AAW sutures demonstrated greater strength. This may have been influenced by the unevenness of the mechanical properties of the sutures, which may be indirectly indicated by the largest variation in maximum load to failure for the intact Maxon^TM^ specimens (28.9 to 39.1 N). Although Novafil^TM^ is a non-absorbable monofilament material, a notable distinction was observed between the breaking strength of the suture implanted under the skin on the withers and the intact suture 3 weeks following the implantation (14.69 ± 3.54 vs. 20.71 ± 0.87, *p* = 0.006). This could also be explained by the uneven mechanical properties of different areas of the suture, since the suturing of the AAW and the implantation under the skin were performed with a suture from the same blister.

### 3.4. Histology

**Figure 8:**
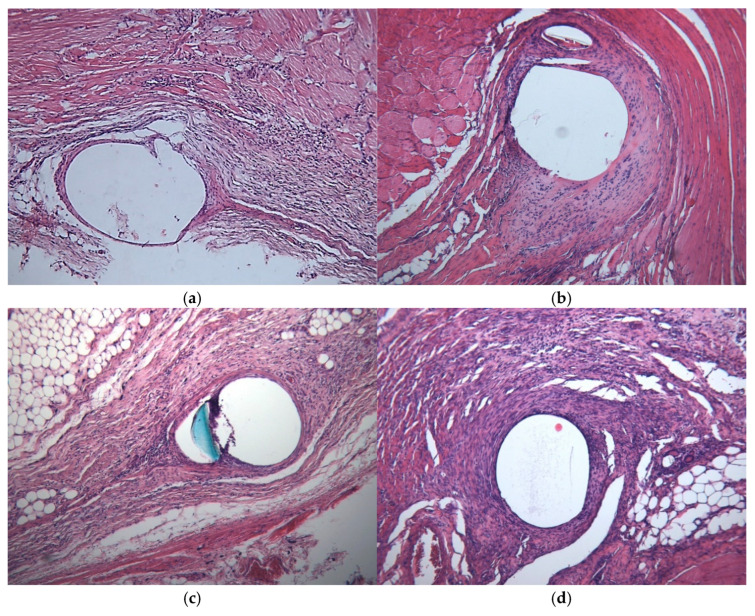

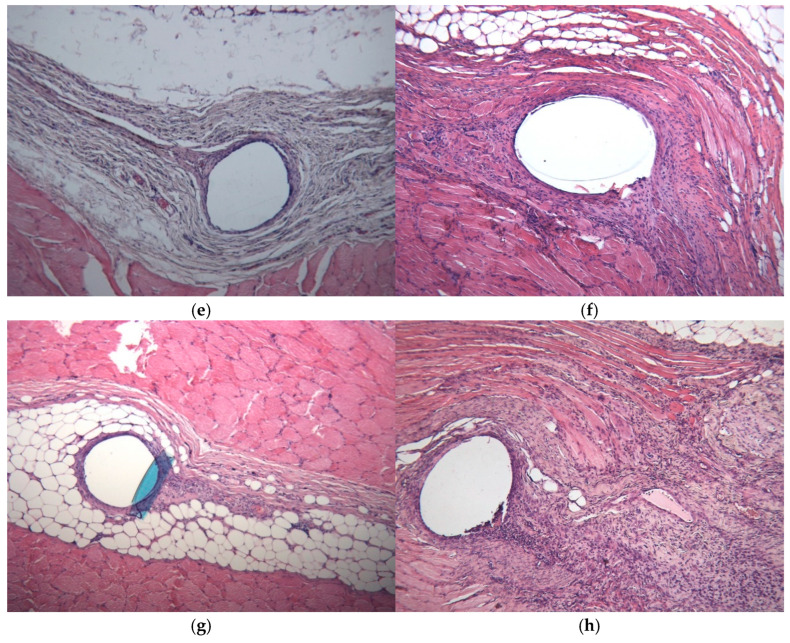
Representative tissue reaction to implanted suture material at 3 weeks post-implantation ((**a**,**b**), V-loc^TM^ 180; (**c**,**d**), V-loc^TM^ PBT; (**e**,**f**), Maxon^TM^; (**g**,**h**), Novafil^TM^) depending on site ((**a**,**c**,**e**,**g**), back; (**b**,**d**,**f**,**h**), abdominal wall). Hematoxylin-eosin, ×10.

All the sutures extracted from the subcutaneous pocket at the withers 3 and 6 weeks following the implantation were surrounded with a thin layer of formed connective tissue. The cellular infiltrate consisted mainly of lymphocytes. Average tissue reaction scores did not differ between the groups (Table 5). The maximum tissue reaction was observed 3 weeks following the surgical procedure in the vicinity of the sutures utilized to close the myofascial defect. In nearly all groups, the sutures were encased in unformed connective tissue, accompanied by a notable lymphocytic infiltration and moderate macrophage reaction. No statistically significant differences were identified between the groups, yet the degree of the tissue reaction severity was markedly elevated in comparison to the tissue reaction to the suture implanted on the withers across all groups (Table 5, Figure 8).

**Figure 9:**
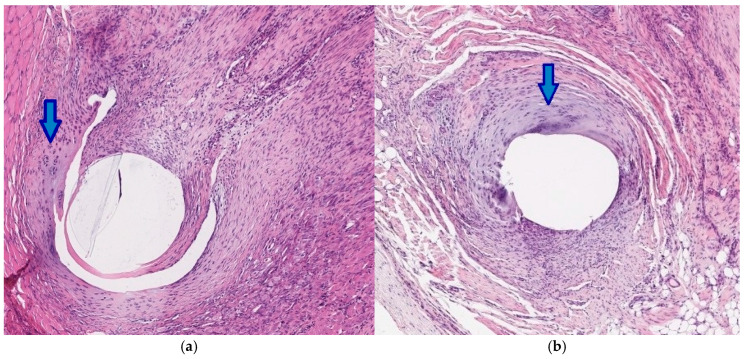
The mature chondrocytes (arrows) around a unidirectional absorbable V-loc 180 suture 3 weeks (**a**) and 6 weeks (**b**) following the implantation in the abdominal wall. Hematoxylin-eosin, ×10.

Six weeks following the procedure, the average tissue reaction scores decreased significantly in all groups, not differing from the reaction at the withers. In two animals from the V-loc^TM^ 180 group, a violation of the connective tissue formation was noted at 3 and 6 weeks post-implantation, as evidenced by the appearance of mature chondrocytes around the suture (Figure 9).

## 4. Discussion

One of the primary objectives of surgical intervention for postoperative hernias is to attempt to restore the anatomy and function of the abdominal wall. Consequently, the closure of the fascial defect is regarded as a crucial aspect of both open and laparoscopic hernia repair. Accordingly, for patients undergoing midline postoperative hernia repair (laparoscopic or open), the European Hernia Society (EHS) recommends closing the fascial defect and avoiding a bridging technique with surgical mesh [22]. According to the data published by Arias-Espinosa, L. et al. [23], 93.4% (*n* = 10,527) of patients in the Abdominal Core Health Quality Collaborative database registry underwent fascial closure for their ventral hernia repair. Overall, about 40% of repairs in the study involved barbed sutures. In the treatment of rectus diastasis, which often accompanies midline ventral hernias, the EHS identifies two main surgical treatment options which are also associated with suturing the linea alba: linea alba plication (suture) without mesh augmentation and linea alba plication with mesh augmentation, both by an open or laparoendoscopic approach [24].

Different types of sutures (absorbable, non-absorbable, simple, or barbed) and different suture techniques (running, interrupted, single, or double layer) were described for this purpose; however, no definitive recommendation on the type of suture or suturing technique can be made [24]. The recent literature failed to identify any randomized controlled studies comparing the commercially available barbed monofilament wound closure devices, neither absorbable nor non-absorbable, for primary fascial closure, and information about the structural and mechanical properties of various types of suture materials is available mainly from manufacturers’ descriptions. A recent systematic review included 62 articles with low-to-moderate quality, including 2158 samples from 10 different animal species across 27 surgical procedures. The findings indicated that barbed sutures exhibited a significant reduction in suture time, limited change in the Cross-Sectional Area, and decreased instances of tissue disruption. However, subgroup analyses, considering both clinical and research significance, indicated that barbed sutures might cause more specific adverse events and demonstrate a suboptimal performance of physical properties/reliability [25].

Therefore, we selected four materials for examination, two of which were absorbable, and two were non-absorbable. It was these types of suture materials that demonstrated advantages in suturing a linea alba defect, compared with a rapidly absorbable material, the use of which led to recurrence in 40% of cases [26]. The absorbable sutures were composed of a single type of polymer, while differing in the polymer structure: smooth monofilament (Maxon^TM^) and unidirectional monofilament (V-loc^TM^ 180). The non-absorbable sutures were also presented as a smooth monofilament (Novafil^TM^) and a unidirectional monofilament (V-loc PBT^TM^) with the same chemical composition.

In addition, all the sutures were implanted in animals under two different conditions: as a suture under tension and as a material under the skin. This was performed to evaluate the behavior of the polymer and the reaction of surrounding tissues to it. As a model, a myofascial defect of the anterior abdominal wall (AAW) was created, which was then sutured with a continuous small bite suture using one of the investigated materials. The results demonstrated that, regardless of the type of suture, both materials implanted into the subcutaneous pocket at the withers resulted in the formation of a connective tissue capsule around the sutures by 3 weeks following the procedure, with a minimally pronounced tissue reaction. The inflammatory reaction to all sutures during the suturing of the myofascial defect 3 weeks following the procedure was maximal and significantly different from the reaction to subcutaneous implantation. Zaruby J et al. [21] have also demonstrated the maximum tissue reaction 21 days following the suturing of a skin wound of minipigs by two commercially available barbed suture devices and one monofilament suture: the size 4-0 V-Loc^TM^ 90, the size 3-0 Quill Monoderm^®^, and the size 4-0 Biosyn^TM^ devices. In contrast to the earlier timeframe, the authors did not find any significant difference in the grade of tissue reaction between the groups, as we did in our study. Law AY et al. [27] sutured a skin wound in dogs with a rapidly absorbable unidirectional or smooth suture material having the same polymer composition (3-0 Glycomer^TM^ 631 barbed suture V-LOC^TM^ and non-barbed 3-0 Glycomer^TM^ 631 suture Biosyn^TM^) and demonstrated significantly higher cellular infiltrate, fibrosis, neovascularization, and average tissue reaction scores for barbed sutures compared to non-barbed ones. This may be attributed to an earlier examination period (14 days) and the use of rapidly absorbable materials.

Six weeks following the procedure, the tissue reaction did not depend on the type of sutures or the place of implantation. However, four rats (40%) from the Maxon^TM^ group demonstrated the suture failure of the myofascial defect and the formation of diastasis. The group of unidirectional sutures from the same V-Loc^TM^ 180 polymer did not exhibit any instances of suture failure. However, histological examination revealed the presence of mature chondrocytes in the vicinity of the sutures in one animal at three weeks and another at six weeks following the procedure. It can be reasonably inferred that the inflammatory response to the absorbable polymer impedes the normal wound-healing process. The observed differences in the type of response may be related to the specific type of suture material. Hoer et al. demonstrated that distinct suture closure techniques were associated with varying concentrations of collagen protein and Type I/III collagen ratio [28]. Furthermore, the group have also demonstrated that high-tension closures negatively impacted the abdominal wall perfusion in a small animal model [29]. In addition, Kushner BS et al. observed a statistically significant increase in necrosis at the incision line 1 week following the closure with a small bit Polydioxanone (PDS) II suture as compared with barbed STRATAFIX^TM^ Symmetric PDS^TM^ Plus Device [30].

An in vitro degradation profile of a barbed suture sample was studied by Hong W. et al. [31]. Results of this study demonstrated that though the scanning electron microscopy image of the barbed sutures immersed in a serum bottle containing phosphate-buffered saline for 7 days (T7) and 14 days (T14) did not show any obvious damage on the surface, the tensile strength of the threads was decreased after immersion. Next, the pull-out strength experiments developed in this study were performed. The average maximum holding capacity of intact barbs was significantly higher than T7 and T14 (T0 vs. T7, *p* < 0.001; T0 vs. T14, *p* < 0.001). The authors suggested the missing information on barbed sutures regarding mechanical properties may produce unknown risks for patients.

The findings pertaining to the structural and mechanical properties of sutures obtained within our study are noteworthy. Despite the fact that we used all sutures of the same dimension (USP 3-0), their diameter significantly differed from each other, which probably determined the differences in the strength of intact sutures. Li S. et al. [32] assessed the biomechanical differences between USP 2-0, 3-0, and 4-0 PGA sutures using uniaxial tensile testing on canine midline skin and fascial muscle tissues; they analyzed tissue reactivity and mRNA and protein expression levels of inflammatory factors and demonstrated that larger diameter sutures led to increased levels of inflammatory factors (IL-1β, IL-6, and TNF-α) and tissue reactivity. In this work, there were no statistically significant differences observed in the maximum tensile strength among different sizes of sutures according to the data of biomechanical testing in muscles, but larger-diameter sutures consistently showed higher levels of inflammatory factors and tissue reactions at various time points. The authors concluded that despite the fact that all suture sizes completely degraded in the muscle after 3 months of suturing, the impact of different suture sizes on the early postoperative tissue reaction cannot be ignored.

In addition, our data on the loss of strength do not match the data proposed by the manufacturers. According to their data, in the first 2 weeks following the implantation of the suture material, it retains approximately 75% of the strength, in 3 weeks 65%, in 4 weeks 50%, and in the 6th week 25%. Our data show that the preserved strength of the V-loc^TM^ 180 utilized in the myofascial defect suture 3 weeks following the procedure was 92% (ranging from 86 to 94%), and for implantation under the skin it was 93% (ranging from 86 to 96%). For Maxon^TM^, the preserved strength at the myofascial defect suture 3 weeks post-procedure was 105% (ranging from 91 to 114%), and at the subcutaneous implantation site it was 93% (ranging from 76 to 106%). By week 6 post-procedure, the preserved strength of the V-loc^TM^ 180 sutures at the myofascial defect was 33% (ranging from 15 to 58%), and that of sutures implanted under the skin was 49.7% (ranging from 48 to 59%). For Maxon^TM^, these values were 38% (35–48%) for the suturing defect and 44% (34–49%) for the subcutaneous implantation.

Wound healing following laparotomy is characterized by a complex and predictable cascade of events: the immediate migration and marginalization of inflammatory cells with the ultimate release of cytokines to increase vascular permeability, to promote vascular dilation, and to increase blood flow [33]. It is therefore possible that a long-lasting, sufficiently large volume of absorbable material, which attracts active phagocytic cells, may sustain inflammation and interfere with the formation of scar tissue.

All the studied suture materials demonstrated high tissue compatibility when implanted on the withers. However, in wound healing under conditions requiring the suture tension for the wound edges approximation, the reaction to the significant amount of absorbable polymer may change, especially in patients with additional risk factors (obesity, impaired collagen synthesis, and immunosuppression), which may contribute to the development of wound complications and an increased recurrence rate. Additionally, one of the major complications of impaired wound healing can be surgical site infection (SSI). With regard to suture material, recent studies have clearly shown that the addition of triclosan to suture material can significantly reduce SSI [34]. Triclosan forms an inhibition zone around the suture material and is effective against the most common pathogens of SSI, especially G-positive bacteria. The use of triclosan-coated barbed sutures (Stratafix Symmetric, Johnson & Johnson, New Brunswick, NJ, USA) and triclosan-coated polydioxanone loop sutures (PDSs) (Johnson & Johnson) for aponeurotic closure in emergent surgery reduces the incidence of incisional SSIs [35]. Moreover, triclosan-coated barbed sutures were independently associated with decreased fascial dehiscence rates after emergency laparotomy [36]. According to the results of a current meta-analysis, the use of triclosan-coated sutures for fascial closure statistically significantly reduces the incidence of SSI after abdominal surgery with a risk difference of about 2% [37].

Nonabsorbable barbed sutures appear to be growing in popularity as a novel material for tissue fixation and approximation across different surgical specialties [6,7,8]. In our study, this type of material exhibited an absence of myofascial defect suture failure, sufficient strength for tissue approximation against the background of a moderately pronounced tissue reaction, and the maximum thickness of the scar after 6 weeks.

### Study Limitations

This study was limited by a 6-week follow-up period. A more complete picture of the potential complications may be obtained after a longer observation interval, once the complete degradation of the absorbable material has occurred.

The biomechanical tests of Maxon^TM^ sutures yielded unexpected results. While not statistically significant, the strength of the sutures demonstrated an increase three weeks following the myofascial defect suturing, contrasting with the sutures implanted under the skin. This discrepancy may be attributed to the heterogeneous strength characteristics of the sutures. In order to obtain more accurate data, it might be essential to utilize sutures from disparate blisters during implantation and to assess intact sutures, dividing each into a greater number of test specimens. The tissue response between the study groups was found to be similar; however, complications related to the wound-healing process were observed in the groups where the absorbable material was used. In future studies, it may be necessary to identify various types of collagen and plan immunohistochemical studies of the resulting scar at different stages of wound healing.

## 5. Conclusions

The timing of the biodegradation of suture material may vary depending on the conditions of implantation, which affect the severity of the tissue reaction. Further experimental study is required to elucidate this fact. Absorbable and non-absorbable suture materials have different structural and mechanical properties, and, depending on the conditions and timing of implantation, cause various tissue reactions which affect the wound healing and the number of postoperative complications.

Prior to practical application, surgical sutures should be tested on experimental models that closely resemble the intraoperative conditions in which they will be used.

## Figures and Tables

**Table 1:** Histologic categories for tissue reaction scoring.

Histologic Category	Scoring System
Degree of necrosis	0–4
Congestion/edema	0–4
Cellular infiltrate	0–4 *
Neutrophils	
Lymphocytes	
Plasma cells	
Macrophages	
Eosinophils	
Giant cells	0–4
Fibrosis	0–4
Neovascularity	0–4
Calcification	0–4
Fatty infiltration	0–4
Foreign body reaction	0/1

* 0, entity not present, 1, entity present to a very mild degree (occasional), 2, entity present to a mild degree, 3, entity present to a moderate degree, and 4, entity present to a marked degree. *—Number of cells scored by average of four fields at ×45: 0, 0 cells; 1, 1–5 cells; 2, 6–15 cells; 3, 16–25 cells; and 4, >25 cells [21].

**Table 2:** Thickness of postoperative scar.

	V-Loc^TM^ 180	V-Loc^TM^ PBT	Maxon^TM^	Novafil^TM^
Thickness, mm 3 weeks	2.52 ± 0.24	2.38 ± 0.22	2.37 ± 0.21	2.24 ± 0.43
Thickness, mm 6 weeks	3.18 ± 0.43	3.42 ± 0.15	2.43 ± 0.76	2.57 ± 0.23
*p*	0.028	0.008	1	0.291

**Table 3:** Breaking strength of intact suture material samples.

Type of Suture Material	Maximum Load to Failure, N Mean ± SD (Min–Max)	*p*
V-Loc^TM^ 180	37.02 ± 0.42 (36.21–37.44)	<0.001 vs. V-loc^TM^ PBT <0.01 vs. Maxon^TM^ <0.001 vs. Novafil^TM^
V-Loc^TM^ PBT	21.31 ± 1.14 (19.59–23.05)	<0.001 vs. V-loc^TM^ 180 <0.001 vs. Maxon^TM^
Maxon^TM^	33.61 ± 3.04 (28.88–39.10)	<0.01 vs. V-loc^TM^ 180 <0.001 vs. V-loc^TM^ PBT <0.001 vs. Novafil^TM^
Novafil^TM^	20.71 ± 0.87 (19.47–22.13)	<0.001 vs. V-loc^TM^ 180 <0.001 vs. Maxon^TM^

**Table 4:** The mean load to failure of the studied sutures depends on the time and place of implantation.

Time	Group/Place	Abdominal Wall	Back	*p*
3 weeks	V-loc^TM^ 180	33.86 + 1.05	34.41 + 1.07	0.997
	V-loc^TM^ PBT	23.20 + 1.82	21.69 + 1.37	0.678
	Maxon^TM^	36.45 + 4.13	31.54 + 3.75	0.245
	Novafil^TM^	17.59 + 4.22	14.69 + 3.54	0.371
6 weeks	V-loc^TM^ 180	13.52 + 5.45	19.18 + 1.80	0.005
	V-loc^TM^ PBT	20.94 + 3.06	21.51 + 1.36	0.987
	Maxon^TM^	13.39 + 1.60	14.44 + 2.22	0.993
	Novafil^TM^	19.21 + 2.71	19.57 + 1.19	0.999

**Table 5:** The average tissue reaction scores to the suture material depending on the place and timing of implantation.

Time	Group/Place	Abdominal Wall	Back	*p*
3 weeks	V-loc^TM^ 180	0.79 [0.67–0.96]	0.34 [0.25–0.50]	0.028
	V-loc^TM^ PBT	0.83 [0.63–0.88]	0.25 [0.21–0.46]	0.041
	Maxon^TM^	0.83 [0.75–0.92]	0.42 [0.29–0.54]	0.021
	Novafil^TM^	1.0 [0.84–1.64]	0.29 [0.21–0.50]	0.021
*p*		0.483	0.814	
6 weeks	V-loc^TM^ 180	0.42 [0.34–0.46] *	0.42 [0.25–0.58]	0.859
	V-loc^TM^ PBT	0.33 [0.29–0.42] *	0.54 [0.46–0.63]	0.061
	Maxon^TM^	0.29 [0.17–0.54] *	0.42 [0.42–0.50]	0.289
	Novafil^TM^	0.21 [0.13–0.29] *	0.25 [0.17–0.50]	0.479
*p*		0.252	0.281	

*—*p* < 0.05 vs. 3 weeks.

## Data Availability

The raw data supporting the conclusions of this article will be made available by the authors on request.
